# Health Impacts and Biomarkers of Prenatal Exposure to Methylmercury: Lessons from Minamata, Japan

**DOI:** 10.3390/toxics6030045

**Published:** 2018-08-03

**Authors:** Mineshi Sakamoto, Nozomi Tatsuta, Kimiko Izumo, Phuong Thanh Phan, Loi Duc Vu, Megumi Yamamoto, Masaaki Nakamura, Kunihiko Nakai, Katsuyuki Murata

**Affiliations:** 1 National Institute for Minamata Disease, Minamata, Kumamoto 867-0008, Japan; izumo@nimd.go.jp (K.I.); yamamoto@nimd.go.jp (M.Y.); nakamura@nimd.go.jp (M.N.); 2 Development and Environmental Medicine, Tohoku University Graduate School of Medicine, Miyagi 980-8575, Japan; nozomi@med.tohoku.ac.jp (N.T.); winestem@med.akita-u.ac.jp (K.N.); 3 Faculty of Chemistry, Thai Nguyen University of Sciences, Thai Nguyen 250000, Vietnam; phuongqtdhkh@gmail.com; 4 Institute of Chemistry, Vietnam Academy of Science and Technology, Hanoi 100000, Vietnam; ducloi@ich.vast.vn; 5 Department of Environmental Health Sciences, Akita University School of Medicine, Akita 010-8543, Japan; satoc@med.tohoku.ac.jp

**Keywords:** methylmercury, kinetics, toxicity, fetus, exposure assessment

## Abstract

The main chemical forms of mercury are elemental mercury, inorganic divalent mercury, and methylmercury, which are metabolized in different ways and have differing toxic effects in humans. Among the various chemical forms of mercury, methylmercury is known to be particularly neurotoxic, and was identified as the cause of Minamata disease. It bioaccumulates in fish and shellfish via aquatic food webs, and fish and sea mammals at high trophic levels exhibit high mercury concentrations. Most human methylmercury exposure occurs through seafood consumption. Methylmercury easily penetrates the blood-brain barrier and so can affect the nervous system. Fetuses are known to be at particularly high risk of methylmercury exposure. In this review, we summarize the health effects and exposure assessment of methylmercury as follows: (1) methylmercury toxicity, (2) history and background of Minamata disease, (3) methylmercury pollution in the Minamata area according to analyses of preserved umbilical cords, (4) changes in the sex ratio in Minamata area, (5) neuropathology in fetuses, (6) kinetics of methylmercury in fetuses, (7) exposure assessment in fetuses.

## 1. Methylmercury Toxicity

Mercury has been used by humans for centuries due to its unique physical and chemical properties. However, the different chemical forms of mercury, which include elemental mercury (Hg^0^), inorganic divalent mercury (Hg^2+^), and organic mercury (mainly methylmercury, CH_3_Hg^+^), cause a variety of toxic effects [1,2]. Differences in exposure sources, the affected organs, toxic effects, and metabolism are seen among the various chemical forms of mercury. For example, the general population is exposed to small amounts of elemental mercury due to its use in dental amalgams. On the other hand, workers at artisanal small-scale gold mining sites and gold shops in Amazon River regions can be exposed to high levels of elemental mercury, as they often have to heat gold-mercury amalgams to evaporate the mercury and obtain the gold [2]. In humans, minimal amounts of liquid elemental mercury are absorbed from the digestive tract, and so liquid elemental mercury does not cause acute toxicity, even when the liquid mercury in a thermometer is accidentally ingested. However, problems can arise when liquid mercury is heated and bursts into the surrounding air [2]. A large proportion of inhaled gaseous elemental mercury (approximately 80%) is absorbed into the blood via the lungs, and, as an uncharged and therefore lipid-soluble substance, it can easily pass through the blood-brain barrier. With time, the gaseous elemental mercury in the patient’s brain is oxidized to inorganic divalent mercury and causes damage to the brain, and the inorganic mercury also accumulates in the kidneys, where it causes renal damage [2]. The amount of inorganic mercury absorbed through the digestive tract is comparatively low (5–10%) [3]. However, the intake of a large amount of inorganic mercury compounds, such as mercury (II) chloride, in cases of accidental ingestion or ingestion with suicidal intent causes digestive tract and kidney disorders, which can result in death [3]. In the environment, a part of the divalent mercury can be changed to methylmercury by some micro-organisms and sunlight. It then bioaccumulates in fish and marine mammals which exhibit elevated, food-web dependent methylmercury levels [4]. Most human methylmercury exposure occurs from the consumption of fish and seafood. Methylmercury is readily absorbed by the digestive tract (>90% is absorbed) [5]. In addition, it exhibits high affinity for sulfhydryl groups [1], and some methylmercury combines with L-type cysteine to form l-cysteine-methylmercury conjugates, which have similar chemical structures to methionine, an essential amino acid [6]. The conjugates are then distributed to all tissues, including the brain (via the blood brain barrier), as they are treated like L-type neutral amino acids [7,8]. In the epidemics of marked methylmercury intoxications in Minamata [9,10], Japan and Iraq [11], the brain was the organ that was most severely affected, particularly in that the developing brains of fetuses were damaged [12,13]. 

## 2. History and Background of Minamata Disease

The epidemic of methylmercury intoxication that occurred in Minamata area, Kumamoto Prefecture, Japan, is known as “Minamata disease”. It was the first experience of severe methylmercury poisoning caused by anthropogenic environmental pollution [10]. The causative agent, methylmercury, was produced from inorganic mercury as a byproduct of the process used to manufacture acetaldehyde at Chisso Co., Ltd. (Kumamoto, Japan), in Minamata City, and it was directly discharged into Minamata Bay [10]. People who consumed a large amount of fish and shellfish that had been contaminated with methylmercury from Minamata Bay developed symptoms of methylmercury toxicity. The first patient to suffer from neurological symptoms was reported in May 1956. Kumamoto University soon started to investigate the cause of this outbreak, and in March 1957 they reported that they suspected that this disease was a type of heavy metal poisoning transmitted via fish and shellfish consumption [10]. However, because this was the first case of methylmercury poisoning due to environmental pollution it took many years for the cause and effect relationship to be fully elucidated. In the meantime, the number of patients with Minamata disease started to rapidly increase, mainly in the fishing village of Minamata [10]. Therefore, people were afraid that the disease was “a strange contagious disease” with an unknown cause. In 1958, Chisso Co., Ltd., moved their effluent outlet from Minamata Bay to the Minamata River in an effort to ameliorate the epidemic taking place in the areas near Minamata. However, this resulted in the disease spreading to the areas surrounding Minamata City. In addition, a second epidemic of methylmercury poisoning, so-called “Niigata Minamata disease”, occurred in 1965. This outbreak was caused by methylmercury derived from the same acetaldehyde production process as was responsible for the epidemic in Minamata City. Chisso Co., Ltd. stopped acetaldehyde production in May 1969. In September 1969, almost 12 years after the first case of Minamata disease was encountered, the Japanese government officially announced that the causative agent of Minamata disease was methylmercury, which had been discharged from the above-mentioned chemical plants. 

## 3. Methylmercury Pollution in the Minamata Area According to Analyses of Preserved Umbilical Cords

**Figure 1:**
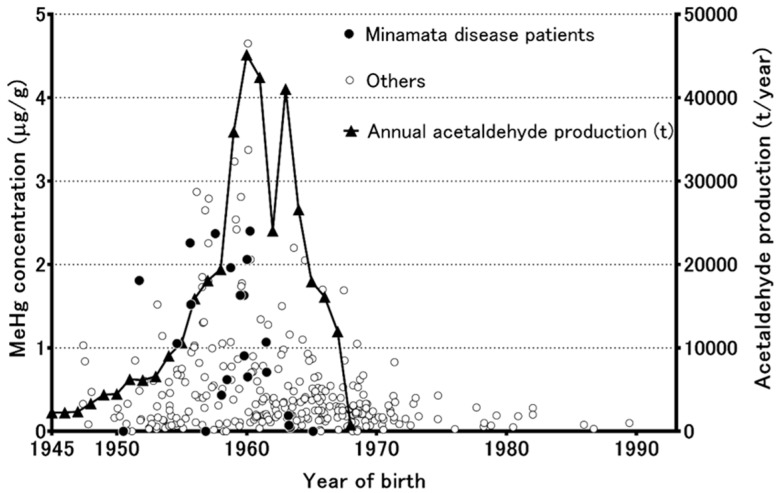
Individual methylmercury concentrations of preserved umbilical cords (μg/g dry weight) from the Minamata area and the amount of annual acetaldehyde production.

Unfortunately, no human or biota samples were collected from the Minamata area during the period of severe methylmercury pollution, and so methylmercury exposure levels could not be determined. Therefore, the time-course and regional distribution of methylmercury pollution in the Minamata area were unknown. However, Japanese people have an ancient custom of preserving their children’s umbilical cords as mementos, and so it was possible to assess methylmercury exposure levels in the Minamata area by analyzing the preserved umbilical cords of children born in the region, which in turn provided a reliable estimate of the time-course of the changes in methylmercury pollution [14,15]. A study by Nishigaki and Harada (1975) achieved a breakthrough in that it revealed that the Minamata inhabitants’ methylmercury exposure levels peaked in the first five years of the 1950s, and then the methylmercury levels of the preserved umbilical cord tissue samples decreased according to the decline of acetaldehyde production in the Minamata area. Sakamoto et al. [16] analyzed a total of 325 umbilical cord samples, including 124 newly collected samples and the 164 samples collected during the studies published by Harada et al. [15,17,18]. Figure 1 shows the methylmercury concentrations of individual preserved umbilical cords (μg/g dry weight) from the Minamata area and the annual level of acetaldehyde production at the time the samples were collected. Elevated methylmercury concentrations (≥1 μg/g) were mainly observed in the inhabitants born from 1947 to 1968. The peak methylmercury concentrations (≥2 μg/g) were mainly observed during the period from 1955 to 1959, when the typical fetal-type Minamata disease patients were born [13], and a reduction in the frequency of the male sex was detected in the Minamata area by Sakamoto et al. [16]. The residents’ methylmercury concentrations started to decrease along with the decline in acetaldehyde production, which ceased in 1968. After 1968, no individuals with elevated methylmercury concentrations (≥1 μg/g) were encountered. These unique retrospective studies of the methylmercury concentrations of preserved umbilical cord samples revealed not only the historical time-course of methylmercury pollution, but also its regional distribution, in the Minamata area. Sakamoto et al. [19] also calculated a conversion factor for converting methylmercury concentrations in dry weight of cord tissue to the equivalent maternal hair (0–1 cm from the scalp) level (conversion factor: 24.09). Preserved umbilical cord tissue would also be useful for retrospective dose-response studies of methylmercury exposure and the occurrence of fetal-type Minamata disease in Japan.

## 4. Changes in the Sex Ratio in Minamata City

Several decades ago, skewed sex ratios at birth due to hazardous chemicals became a matter of international concern, especially the skewed sex ratio caused by dioxin contamination in Seveso, Italy [20]. As mentioned above, prenatal methylmercury exposure has much stronger effects on fetuses than on their mothers. Therefore, Sakamoto et al. examined the sex ratio at birth in Minamata area to evaluate the effects of severe methylmercury exposure [16]. In four of the five years from 1955 to 1959, unexpectedly low numbers of males were born in Minamata City. Furthermore, a dose-dependent (based on the estimated environmental level of methylmercury) reduction in the frequency of the male sex was observed in that period (male sex:total population ratio; 0.515 for Kumamoto City (the control), >0.492 for Minamata City, >0.459 for the area in which Minamata disease was most prevalent, and >0.382 among the families of fishermen). The lowest male:total population ratio (0.393) was observed among the offspring of mothers with Minamata disease [16]. However, no reduction in the frequency of the male sex was observed in the cases in which only the father was affected by Minamata disease. This phenomenon was suspected to have been caused by maternal methylmercury exposure leading to methylmercury having direct effects on fetuses. In addition, the proportion of male stillbirths in the city increased to 173 males/100 females (male proportion: 0.634) when the methylmercury pollution was at its most severe, indicating that more males were lost at the fetal stage. The increased proportion of stillborn males in Minamata City partly explains the lower proportion of males in the abovementioned study. However, little is known about the sex-related differences in the susceptibility of fetuses against the lethal effects of methylmercury.

## 5. Neuropathology in Fetuses

**Figure 2:**
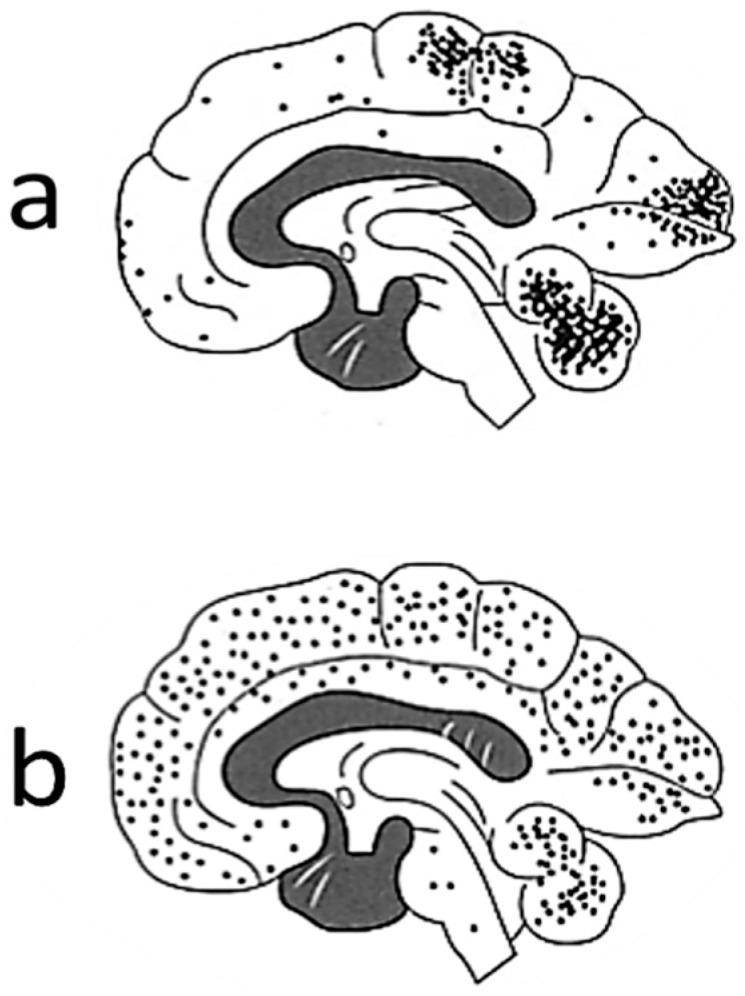
Comparisons of the distributions of lesions among adult and fetal-type cases of Minamata disease. Note: The distributions of degenerated neurons in adult (**a**) and fetal patients (**b**) are shown. (Modified from Reference [9] with permission).

Among the adults with Minamata disease that were exposed to high levels of methylmercury, neuronal degeneration was observed from medical autopsies, predominantly in certain areas of the cerebral cortex (the parietal, occipital, and temporal lobes), cerebellum, and peripheral nerves [9]. Figure 2a shows the lesion distribution among adult cases of Minamata disease. The main symptoms exhibited by these patients included sensory disturbances in the distal parts of the extremities followed by ataxia, concentric contraction of the visual field, impairment of gait and/or speech, muscle weakness, tremors, abnormal eye movement, and hearing impairment [21], which mainly reflected the areas of the brain that suffered nerve damage [9]. In Iraq, a large-scale epidemic of methylmercury poisoning occurred in 1972–1973 after wheat seeds were disinfected with methylmercury [11]. This outbreak affected more than 6000 people and resulted in 400 deaths. The main symptoms were similar to those of Minamata disease. A study conducted in Iraq [11] showed that in adult cases of methylmercury poisoning the estimated mercury body burden thresholds (mg) at diagnosis for various symptoms were as follows: abnormal sensory perception, about 25 mg (equivalent to a mercury blood concentration of 250 μg/L); ataxia, about 50 mg; articulation disorders, about 90 mg; hearing loss, about 180 mg; death, >200 mg.

In Minamata City, a high incidence of cerebral palsy was observed from 1955 to 1959, when the most severe cases of Minamata disease occurred. The incidence rate was 5.8%, which was much higher than the normal incidence rate (0.2–0.64%) in Japan [10]. The study group of Kumamoto University concluded that they were fetal-type Minamata disease patients, who were exposed to methylmercury via placenta [10]. The symptoms observed in fetal-type Minamata disease patients (22 typical severe cases) were mental retardation, inability of walking by oneself, disturbances of coordination, speech, chewing and swallowing, and increased muscle tone, which are similar to the symptoms of cerebral palsy [10]. Histopathological examinations of Japanese fetal-type Minamata disease patients revealed widespread and severe neuronal degeneration in the central nervous system [18]. Figure 1b shows the lesion distribution fetal-type cases of Minamata disease. On the other hand, their mothers had mild or no manifestations of methylmercury poisoning [13]. In Iraq, the children that were most severely affected by methylmercury poisoning manifested with severe sensory impairments, general paralysis, hyperactive reflexes, and/or impaired mental development [11]. The world Health Organization suggested that Iraqi data implied that a peak maternal hair mercury level of 10–20 μg/g is associated with a 5% risk of neurological disorders [5]. 

In an animal experiment using rats, distinct patterns of neuronal degeneration were observed [22] by methylmercury administration at various stages of brain development. For example, neonatal rats administered methylmercury for 10 days from postnatal day (PD) 2 showed minimal damage in the hippocampus and brainstem nuclei, young rats administered 10 days from PD 15 showed neuronal degeneration in the cerebral cortex, striatum, and red nucleus, while adult rats from PD 60 showed severe lesions in the cerebellum and dorsal root ganglia. Moreover, neonatal rats exposed to methylmercury for prolonged period (>30 days) from PD 2 showed the widespread neuronal degeneration in the cerebral neocortex and spinal sensory ganglia [23]. These rat experiments indicated that the distribution pattern of methylmercury-induced brain lesions changes depending on the stage of brain development at which methylmercury exposure occurs, and the widespread neuronal degeneration in human fetal-type Minamata disease could be caused by prolonged methylmercury exposure during brain growth.

## 6. Kinetics of Methylmercury in Fetuses

Fetuses depend on their mothers for nutrients, including amino acids, fatty acids, and vitamins. However, they can also be exposed to methylmercury through the maternal consumption of fish and shellfish. Furthermore, some animal studies have shown suggested that methylmercury is actively transferred from mother to fetus via placental amino acid transport systems [24,25,26]. In humans, it was reported that cord blood contains higher concentrations of methylmercury than maternal blood [27,28,29,30,31]. Especially, Stern and Smith [31] summarized the data of the cord blood/maternal blood methylmercury ratio from 10 reports published from 1975 to 2000. Table 1 shows the total maternal and cord blood mercury concentrations recorded in various study populations from the recent papers which were published after 2016. Although the cord blood/maternal ratio varied from 1.03 to 2.04, all the data indicated that cord blood mercury levels were higher than those of maternal blood as summarized by Stern and Smith [31].

In developing fetuses, the brain is sensitive to methylmercury exposure [5]. Both the high sensitivity of the developing brain to methylmercury [38] and the increased methylmercury accumulation in the blood and brain [26] of fetuses are recognized toxicological features of methylmercury. Consequently, the effects of dietary seafood intake in pregnant women remain an important public health issue, especially in populations that consume large quantities of fish and sea mammals, such as toothed whales and seals. Mercury levels in pregnant women can be affected by fish consumption patterns such as the amount, species, frequency, and seasons of the fish consumption during pregnancy. Consequently, the comparison among studies is not always easy [39]. 

## 7. Exposure Assessment in Fetuses

Since the brain is the organ that is most at risk from methylmercury, the biomarkers used to determine human methylmercury exposure levels should reflect the methylmercury concentration in the brain. In humans, methylmercury has an average biological half-life of approximately 70 days (whole body) [5]. Generally, the amount retained in the body reaches an equilibrium during constant methylmercury intake, e.g., from seafood consumption. Animal experiments have indicated that the ratio of the blood mercury concentration to the brain mercury concentration remains constant under steady state conditions. Therefore, the mercury concentration in the blood/red blood cells is a good biomarker for assessing methylmercury exposure [5]. The mercury concentration in hair reflects the blood methylmercury concentration during hair formation and is frequently used as a biomarker for evaluating methylmercury exposure [5]. Although analyses of hair mercury concentrations are affected by a number of variables, such as the hair’s growth rate, density, color, waving, external contamination, and any permanent treatments [5], segmental analysis of maternal hair is able to provide time-course information because the average hair growth rate is commonly assumed to be about 1 cm per month [40,41]. On the other hand, cord blood circulates in the fetal body and can directly reflect the methylmercury concentrations in fetal organs, including the fetal brain, at birth [41]. In addition, a number of studies have employed toenail and/or fingernail mercury concentrations as biomarkers for assessing methylmercury exposure [42,43,44,45]. In most of these studies, toenails rather than fingernails were preferred, because toenails are often less contaminated than fingernails, especially among dental personnel and gold miners, who handle mercury amalgams [2].

**Figure 3:**
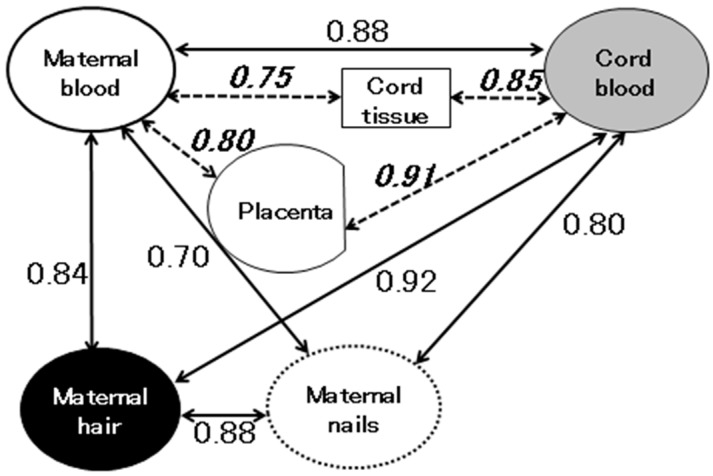
Correlation coefficients (r) for the relationships among biomarkers of methylmercury exposure at parturition. Note: Red blood cells were used to calculate the correlations between the levels of methylmercury in blood, the placenta, and cord tissue (---) [30]. Whale blood was used to calculate the correlations between the methylmercury levels of blood, hair, and nails (**―**) [45].

The organ that is most affected by methylmercury exposure during gestation is the fetal brain. For this reason, biomarkers that reflect fetal methylmercury exposure during gestation are very important for predicting the effects of methylmercury on child development. In a study conducted in the Faroe Islands, the cord blood mercury concentration was the preferred biomarker for evaluating methylmercury exposure, whereas in a study carried out in the Seychelles, the maternal hair mercury concentration was used as the only biomarker of fetal exposure. Umbilical cord tissue has also been used to determine fetal methylmercury exposure levels in some studies [14,46]. In addition, maternal mercury levels in fingernails and toenails at parturition showed strong correlations with those in cord blood [45]. Figure 3 shows the correlation coefficients (r) for the relationships among various biomarkers of methylmercury exposure at parturition, which were obtained from our previous studies [30,45]. All of the biomarkers, including maternal blood, maternal hair, cord blood, maternal nails, the placenta, and cord tissue showed strong correlations with each other (r: >0.70). This suggests that all of the examined biomarkers are useful for assessing the prenatal exposure of fetuses to methylmercury. 

## 8. Summary

The United Nations Environment Programme (UNEP) agreed to develop a global legally-binding instrument on Hg in 2013 as the Minamata Convention on Mercury [4], and the treaty entered into force in August of 2017. UNEP concluded that the rapid increase in historical environmental Hg levels began during the industrial revolution in the 19th century. The impact of increased Hg will appear as increased methylmercury levels in the marine environment, especially in fish and marine mammals, and finally in humans who consume marine products. Among the human populations, fetuses who bear the next generation are at the highest risk to the methylmercury exposure. Therefore, we need an effort to reduce the anthropogenic emissions/releases of mercury into the environment, “recognizing to reflect the lessons learned from Minamata disease”.

## Figures and Tables

**Table 1:** Total maternal and cord blood mercury concentrations in various study populations.

Study Site	Measure		Maternal Blood Hg	Cord Blood Hg	Cord Blood/Maternal Blood Ratio	Sampling Years and (Published Year) References
Ten cites, Canada	T-Hg	μg/L	0.562 (*n* = 1673)	0.802 (*n* = 1419)	1.43	2008–2011 (2016) [32]
Laizhou By, China	T-Hg	μg/L	0.72 (*n* = 410)	1.20 (*n* = 410)	1.67	2010–2012 (2016) [33]
Busan, Korea	T-Hg	μg/L	3.12 (*n* = 127)	5.46 (*n* = 127)	1.75	2009–2010 (2016) [34]
Tong Gang, Taiwan	T-Hg	μg/L	2.24 (*n* = 145)	2.30 (*n* = 145)	1.03	2010–2011 (2017) [35]
Tokyo, Japan	T-Hg	μg/L	4.97 (*n* = 334)	10.15 (*n* = 334)	2.04	2010–2012 (2018) [36]
Kumamoto, Japan	T-Hg	ng/g	3.79 (*n* = 54)	7.26 (*n* = 54)	1.92	2006–2007 (2018) [37]
